# Clinical profiles and risk factors for outcomes in older patients with cervical and trochanteric hip fracture: similarities and differences

**DOI:** 10.1186/1752-2897-6-2

**Published:** 2012-02-15

**Authors:** Alexander A Fisher, Wichat Srikusalanukul, Michael W Davis, Paul N Smith

**Affiliations:** 1 Department of Geriatric Medicine, The Canberra Hospital, PO Box 11, Woden, ACT 2606, Australia; 2 Department of Orthopaedic Surgery, The Canberra Hospital, PO Box 11, Woden, ACT 2606, Australia; 3 The Canberra Hospital, Australian National University Medical School, Canberra, ACT 2606, Australia

**Keywords:** Hip fracture type, Clinical characteristics, Predictors of outcomes

## Abstract

**Background:**

Data on clinical characteristics and outcomes in regard to hip fracture (HF) type are controversial. This study aimed to evaluate whether clinical and laboratory predictors of poorer outcomes differ by HF type.

**Methods:**

Prospective evaluation of 761 consecutively admitted patients (mean age 82.3 ± 8.8 years; 74.9% women) with low-trauma non-pathological HF. Clinical characteristics and short-term outcomes were recorded. Haematological, renal, liver and thyroid status, C-reactive protein, cardiac troponin I, serum 25(OH) vitamin D, PTH, leptin, adiponectin and resistin were determined.

**Results:**

The cervical compared to the tronchanteric HF group was younger, have higher mean haemoglobin, albumin, adiponectin and resistin and lower PTH levels (all P < 0.05). In-hospital mortality, length of hospital stay (LOS), incidence of post-operative myocardial injury and need of institutionalisation were similar in both groups. Multivariate analysis revealed as independent predictors for in-hospital death in patient with cervical HF male sex, hyperparathyroidism and lower leptin levels, while in patients with trochanteric HF only hyperparathyroidism; for post-operative myocardial injury dementia, smoking and renal impairment in the former group and coronary artery disease (CAD), hyperparathyroidism and hypoleptinaemia in the latter; for LOS > 20 days CAD, and age > 75 years and hyperparathyroidism, respectively. Need of institutionalisation was predicted by age > 75 years and dementia in both groups and also by hypovitaminosis D in the cervical and by hyperparathyroidism in the trochanteric HF.

**Conclusions:**

Clinical characteristics and incidence of poorer short-term outcomes in the two main HF types are rather similar but risk factors for certain outcomes are site-specific reflecting differences in underlying mechanisms.

## Introduction

In elderly people, hip fractures (HFs) are the commonest cause for acute orthopaedic admissions [1] and the second leading cause of hospitalisation and prolonged length of stay (LOS) [2,3]. One of the important challenges in the management of HF is to identify patients who are at high risk of poor outcome.

Although HFs dominantly are regarded as homogenous, anatomical types--cervical and trochanteric--differ in bone composition and parameters of proximal femur geometry [4-11], as well as in epidemiological, demographic and clinical characteristics [7,12-20]. It is possible that shared biological mechanisms underlie the site, accompanying comorbidities and risks of postoperative complications and outcomes for each type of HF. Apparently osteoporotic HFs and their outcomes are attributable to complex interactions between multiple factors, however, there may exist some common mechanisms determining specific conditions linked to the HF type. These may be indicators that would enable clinicians to identify patients at risk and provide appropriate management.

Various factors have been reported to affect HF outcomes, but the role of anatomic location and the potential implications for clinical practice have been addressed in only few investigations with conflicting results. Studies comparing cervical and trochanteric HFs often evaluated only some clinical and/or laboratory parameters or selected outcomes. The prevailing view was that patients with trochanteric compared to cervical HF have poorer outcomes [7,15,21,22]. However scientific reports on the association of HF type with pre-existing medical conditions, post-operative complications [23-26], LOS [15,27-30], functional outcomes [27,31], and mortality [7,15,17,22,27,32-38] are controversial.

In numerous studies adipocytokines, relatively novel compounds produced by adipocytes, have been found to link energy homeostasis with bone metabolism and to be associated with chronic cardiovascular, metabolic, renal, immunological and lung diseases, all of which are common in HF patients and may affect outcomes. However, the physiological and pathophysiological roles of adipocytokines in causation of various diseases and in predicting mortality are still not fully understood and reports are contradictory. Data regarding the role of the three most studied adipocytokines, leptin, adiponectin and resistin, in patients with HF are sparse [39-41] and their utility as outcome predictors has not been evaluated.

We hypothesized that an integrated approach combining clinical, routine and novel laboratory variables by HF type will improve understanding and prediction of outcomes in these patients. Considering the potential existence of common site-specific pathophysiological mechanisms, it is important to find out whether the interactions between numerous variables differ by HF type and which of them are independent risk factors that can be used in practice for outcome prediction.

Understanding the relationships between HF type, comorbidities and postsurgical outcomes is important in evaluating the role of pathophysiological mechanisms, and may help in individualising prophylaxis, improving prognosis, treatment planning and providing differentially targeted interventions.

The aims of this study were to evaluate (1) whether demographic, clinical haematologic and biochemical parameters differ by HF type, (2) determine the usefulness of HF type as a predictor of short-term outcomes and (3) investigate whether clinical and laboratory predictors of poorer outcomes differ by HF type.

## Patients and methods

### Study population

In this prospective cohort study, all patients aged ≥ 60 years who were admitted with a low-trauma non-pathological hip fracture to the Canberra Hospital between 2000 and 2006 were included. Of 847 admitted patients 86 (10.1%) were excluded from the analysis because of pathological HF due to primary or metastatic bone cancer, multiple myeloma, Paget's disease or primary hyperparathyroidism.

A detailed clinical history and full physical examination was performed, previous case records reviewed and information on demographic characteristics, pre-fracture residential status, use of walking aid, comorbidities, medications, peri-operative complications, in hospital management and short-term outcomes prospectively recorded. The following 11 chronic conditions were included in the analysis: hypertension, coronary artery disease (CAD), previous myocardial infarction, atrial fibrillation, history of stroke, transient ischaemic attack, dementia, diabetes mellitus, Parkinson's disease, chronic obstructive pulmonary disease, and chronic kidney disease. The pre-operative general physical health status was assessed according to the American Society of Anaesthesiologists (ASA) classification. All patients had operative fracture treatment and followed a similar postoperative protocol with mobilisation out of bed on day one.

The study was performed in accordance with the Declaration of Helsinki, the protocol was approved by the local Human Research Ethics Committee and all patients or their carers gave informed consent.

### Laboratory measurements

All patients underwent a standard battery of laboratory tests prior to surgery usually within 12-24 hours after arrival at the Emergency Department. The routine tests included full blood count, serum electrolytes, creatinine, urea nitrogen, C-reactive protein (CRP), albumin and liver function tests, thyroid stimulatory hormone (TSH), free thyroxine (T_4_), vitamin B_12_, folic acid, iron, ferritin, transferrin, as well as cardiac troponin (cTnI), 25(OH) vitamin D (25(OH)D) and intact PTH. Serum cTnI was determined by a two step chemilumenescent microparticle immunoassay (Chemiflex, Abbott Labs, Mississauga, Ontario, Canada). Serum 25 (OH)D was measured by radioimmunoassay kit (Dia Sorin, Stillwater, MN, USA) and intact PTH by two-site chemiluminescent enzyme-linked immunoassay on DPC Immulite 2000 (Diagnostic Products Corp, Los Angeles CA, USA). In these three methods both the intra--and inter-assay coefficient of variations (CVs) ranged from 2.1% to 12.7%. All other measurements were performed by standard automated laboratory methods. Serum calcium concentrations were corrected for serum albumin. Glomerular filtration rate was estimated (eGFR) [42]. All these analyses were performed on the day of blood sampling. Serum cTnI and CRP levels were also assessed within 24 hours post-operatively and then after if elevated and/or clinically indicated.

For the analyses, hypovitaminosis D was defined as 25(OH) D < 50 nmol/L and vitamin D deficiency as < 25 nmol/L. Secondary hyperparathyroidism was defined as elevated serum PTH (> 6.8 pmol/L, the upper limit of the laboratory reference range). Chronic kidney disease was defined as an eGFR < 60 ml/min1.73 m^2^.

In a subset of 284 patients serum concentrations of leptin, adiponectin and resistin were measured in venous blood collected after an overnight fast usually within 48 hours after arrival at the Emergency Department. The plasma was centrifuged immediately and one serum sample was frozen and stored at -70°C until measurement. All samples were analysed at the same time with commercially available kits of the same lot number according to the manufacturer's protocol, and blind to any clinical information. Serum leptin was measured by enzyme-linked immunosorbent assay (ELISA) kit obtained from Diagnostic System Lab Inc (Webster, TX, USA) and serum concentrators of resistin and adiponectin were measured by human ELISA kits (B-bridge International, Inc, Otsuka Pharmaceuticals, Japan). The intra--and inter-assay CVs for these three variables were 4.6-8.9% and 2.3-10.9% respectively.

### Outcome measures

These included: (1) postoperative myocardial injury as defined by cardiac troponin I rise (> 0.06 μg/L), (2) inflammatory response assessed by marked elevation of CRP (> 100 mg/L), (3) length of hospital stay (LOS), (4) being discharged to a permanent residential care facility (RCF) for persons who were admitted from home and (5) all-cause in-hospital mortality. We also determined whether troponin rise and CRP elevation influenced the three other outcomes.

### Statistical analysis

Continuous variables were reported as means ± SD and categorical variables as percentages. Comparisons between groups were performed using analysis of variance and Student's t-test for continuous variables and χ-^2 ^test for categorical variables. Pearson correlation coefficient with log-transformed data (to achieve normal distribution) was used to study the linear correlation between variables. Univariate and multivariate (both linear and logistic) regression analyses were used to determine the odds ratio (OR) and 95% confidence intervals (CI) for associations between HF-type (dependent variable) and different clinical and laboratory variables; all potential confounding variables with statistical significance ≤ 0.15 on univariate analyses were included in the final multivariate analyses. All reported P values are 2-sided; P-values < 0.05 were considered statistically significant. The data was analysed using the Stata software version 10 (StataCorp, College Station, TX, USA).

## Results

### Hip fracture type and comorbidities

Among the 761 study patients, the mean age was 82.3 ± 8.8 years and 74.9% were women (Table 1). The patients with trochanteric HF were slightly older than those with cervical fracture, but this difference disappeared when the age of females and males was analysed separately. Among patients with trochanteric fracture males were younger than females (p = 0.002). The most prevalent comorbidity was hypertension (44.5%), followed by dementia (29.1%), coronary artery disease (22.8%), cerebrovascular disease (20.9%), type 2 diabetes mellitus (16.3%), atrial fibrillation (13.2%), chronic obstructive pulmonary disease (11.4%) and Parkinson's disease (4.4%). All these diseases were similarly common in patients with cervical and trochanteric HF, except Parkinson's which was more prevalent in the former group (6.3% vs. 1.6%, p = 0.002). The proportion of patients with two or more of the listed comorbidities among both types of HF was also similar (39.1% and 47.2% for cervical and trochantric HF, respectively, p = 0.215). Near 1/3 of all patients were admitted from long-term residential care facilities (RCF). The pre-fracture residence status, mobility (using a walking device) and life-style habits (past and current smoking, use of alcohol) did not differ between groups.

**Table 1:** Demographic and clinical characteristics and short-term outcomes in older patients with hip fracture by anatomical site

Characteristics	Total group (n = 761)	Cervical HF (n = 444)	Trochanteric (n = 317)	P value
Age, years (mean ± SD)	82.3 ± 8.8	81.7 ± 8.2	83.1 ± 9.5	**0.034**

Age, females, years (mean ± SD)	82.6 ± 7.7	81.9 ± 7.8	83.1 ± 7.5	0.143

Age, males, years (mean ± SD)	80.6 ± 8.3	81.5 ± 7.3	79.8 ± 9.1	0.359

Females, %	74.9	74.8	75.1	0.489

Admitted from long-term RCF, %	30.3	31.3	29.3	0.705

ASA score ≥ 3, %	72.9	71.1	75.0	0.497

Coronary artery disease, %	22.8	23.8	21.3	0.435

Previous myocardial infarction, %	5.3	4.8	6.1	0.431

Hypertension, %	44.5	44.8	44.1	0.855

Atrial fibrillation, %	13.2	11.6	15.4	0.136

History of stroke, %	13.5	14.4	12.5	0.643

TIA, %	7.4	7.5	7.3	0.942

Dementia, %	29.1	28.5	29.9	0.670

Diabetes mellitus, %	16.3	17.1	15.5	0.709

COPD, %	11.4	10.2	13.1	0.220

Parkinson's disease, %	4.4	6.3	1.6	**0.002**

Current smoker, %	5.4	5.2	5.7	0.752

Ex-smoker, %	9.5	10.6	8.0	0.218

*Alcohol over user, %	5.5	6.1	4.8	0.431

User of walking device, %	34.5	33.5	36.0	0.476

CRP, mg/L	130.6 ± 82.1	134.9 ± 90.7	126.0 ± 71.7	0.356

Post-operative Tnl > 0.06 μg/L, %	27.0	29.5	24.5	0.343

CRP > 100 mg/L %	61.2	61.6	60.7	0.878

LOS , days (mean ± SD)	22.4 ± 23.4	20.9 ± 22.6	24.3 ± 24.4	0.054

LOS ≥ 20 days, %	31.7	28.3	35.3	0.206

New discharges to long-term RCF, %	49.0	48.1	490	0.787

In-hospital death, %	4.9	5.3	4.2	0.676

*Three or more times per week.

RCF, residential care facility; TIA, transient ischaemic attack; ASA, American Society of Anaesthesiologists; COPD, chronic obstructive pulmonary disease; LOS, length of stay; cTnI, cardiac troponin I; CRP, C-reactive protein; RCF, residential care facility.

### Comparison of laboratory parameters in patients with cervical and trochanteric fracture

The mean values for a wide range of pre-operative haematologic variables as well as for biochemical parameters of liver, renal and thyroid functions in patients with two types of HF were similar (Table 2). Among routine parameters only mean haemoglobin and albumin levels differed significantly, both being slightly lower in patients with trochanteric than cervical HF. Although in the former group the prevalence of anaemia (haemoglobin < 120 g/L, 43.0% vs. 33.6%, p = 0.097) and hypoalbuminaemia (< 33 g/L, 28.2% vs. 27.0%, p = 0.819) was slightly higher, the difference was not significant. This was also true for the prevalence of chronic kidney disease on admission (GFR < 60 ml/min 1.73 m^2^, 43.7% vs. 42.8%, p = 0.876).

**Table 2:** Haematologic and biochemical parameters in older patients with hip fracture by anatomical site

Characteristics	Total group (n = 761)	Cervical HF (n = 444)	Trochanteric HF (n = 317)	P value
Erythrocyte count, x10^12^/L	4.1 ± 0.60	4.1 ± 0.61	4.0 ± 0.59	0.084

Haemoglobin, g/L	125.5 ± 16.6	128.2 ± 16.2	121.8 ± 16.5	**0.000**

Leukocyte count, x10^9^/L	10.6 ± 4.2	10.5 ± 4.1	10.8 ± 4.4	0.430

Lymphocytes count, x10^9^/L	1.29 ± 1.22	1.26 ± 1.47	1.32 ± 0.88	0.632

MCV, fl	90.4 ± 8.16	90.3 ± 6.31	90.5 ± 9.78	0.776

MCH, pg/cell	30.7 ± 2.44	30.5 ± 2.56	30.9 ± 2.29	0.198

MCHC, g/L	340.3 ± 34.2	341.2 ± 46.6	339.3 ± 10.19	0.642

RDW, %	15.2 ± 8.7	15.5 ± 12.0	14.8 ± 1.90	0.518

Iron, μmol/L	5.2 ± 4.33	4.8 ± 3.65	5.7 ± 4.92	0.085

Transferrin, g/L	1.7 ± 0.48	1.7 ± 0.52	1.7 ± 0.42	0.324

Transferrin saturation, %	11.6 ± 8.6	10.7 ± 7.9	12.5 ± 9.3	0.095

Ferritin, μg/L	298.8 ± 273.1	290.4 ± 225.4	307.9 ± 317.0	0.606

Vitamin B_12_, pmol/L	395.9 ± 271.4	410.6 ± 276.9	380.1 ± 265.4	0.3601

Folic acid (serum), nmol/L	26.1 ± 15.6	26.7 ± 16.1	25.4 ± 15.0	0.505

Albumin, g/L	35.3 ± 6.4	36.0 ± 6.5	34.5 ± 6.2	**0.002**

Bilirubin, μmol/L	12.4 ± 7.4	12.9 ± 7.8	11.8 ± 6.9	0.214

ALT, U/L	23.0 ± 42.6	29.9 ± 53.9	21.6 ± 25.5	0.581

ALP, U/L	105.4 ± 80.1	104.3 ± 78.6	106.6 ± 82.0	0.813

GGT, U/L	54.1 ± 95.6	54.6 ± 87.3	53.7 ± 104.0	0.936

Urea, mmol/L	8.8 ± 9.1	8.6 ± 10.1	9.0 ± 7.5	0.531

Creatinine, μmol/L	92.9 ± 49.9	91.0 ± 40.3	95.6 ± 60.8	0.217

eGFR, ml/min ·1·73 m^2^	65.1 ± 23.7	65.8 ± 23.1	64.3 ± 24.4	0.600

TSH, mIU/L	1.5 ± 2.17	1.7 ± 2.76	1.4 ± 27	0.246

T_4_, pmol/L	15.9 ± 3.54	16.1 ± 3.64	15.7 ± 3.43	0.336

Serum calcium*(mmol/L)	2.28 ± 0.13	2.28 ± 0.13	2.27 ± 0.13	0.661

Serum phosphate(mmol/L)	0.94 ± 0.48	0.92 ± 0.29	0.97 ± 0.62	0.447

Serum magnesium(mmol/L)	0.78 ± 0.13	0.76 ± 0.13	0.79 ± 0.12	0.059

25(OH)vitamin D(nmol/L)	37.3 ± 18.0	36.9 ± 18.7	37.6 ± 17.4	0.729

PTH(pmol/L)	6.9 ± 5.6	5.9 ± 3.6	8.0 ± 6.9	**0.001**

Leptin (ng/ml)	18.4 ± 23.17	18.1 ± 21.73	18.8 ± 24.67	0.797

Adiponectin (ng/ml)	17.5 ± 7.35	18.5 ± 7.29	16.3 ± 7.28	**0.019**

Resistin (ng/ml)	18.7 ± 10.46	20.1 ± 10.49	16.9 ± 10.19	**0.014**

Values are mean ± SD.

MCV, mean corpuscular volume; MCHC, mean corpuscular haemoglobin concentration; RDW, red-cell distribution; ALT, alanine aminotransferase; ALP, alkaline phosphatase; GGT, gamma-glutamyltransferase; eGFR, estimated glomerular filtration rate; TSH, thyroid-stimulating hormone; T_4_, total thyroxine; CRP, C-reactive protein (highest post-operative levels). Leptin, adiponectin and resistin were measured in 284 patients (147 with cervical and 137 with trochanteric HF).

All variables reflect data on admission (pre-operatively).

The prevalence of hypovitaminosis D (25(OH) D < 50 nmol/L, 77.8% in cervical vs. 82.1% in trochanteric HF, p = 0.370) and vitamin D deficiency (25(OH) D < 25 nmol/L, 34% vs. 24.6%, respectively, p = 0.086) did not differ significantly between groups. Although 25(OH) D concentrations were similarly low in both groups, secondary hyperparathyroidism (PTH > 6.8 pmol/L) was present in 30.2% of patients with cervical and in 41.3% with trochanteric HF (p = 0.033), and the mean PTH value was significantly higher in the latter group (p = 0.001). Among patients with cervical HF and 25(OH) D < 25 nmol/L, blunted PTH response (PTH within normal limits) was more common than in subjects with trochanteric HF (61.2% vs. 43.8%, p = 0.029). Compared to patients with cervical HF those with trochanteric fracture had significantly lower mean serum levels of adiponectin and resistin and a higher leptin: resistin ratio (1.7 vs. 1.1; p = 0.024). After adjustment for age and sex the prevalence of higher serum adiponectin (> 17.1 ng/ml, median level) and resistin (> 16.3 ng/ml, median level) concentrations in patients with cervical HF was respectively 1.7 (OR = 1.74; 95% CI 1.06-2.86; p = 0.029) and 1.9 (OR = 1.95; 95% CI 1.18-3.20; p = 0.007) fold higher than in patients with trochanteric fracture.

### Correlations by hip fracture type

These analyses revealed similarities and differences associated with the HF type (Table 3). In both cervical and trochanteric HF groups, age correlated positively with PTH, cTnI and adiponectin, and negatively with eGFR, haemoglobin correlated positively with eGFR and eGFR correlated negatively with cTnI. In both groups an inverse correlation between PTH and serum calcium (corrected for albumin) as well as eGFR was also present. 25(OH) D was not correlated with any of the variables. Other correlations were site-specific.

**Table 3:** Significant correlations between laboratory variables, age and length of hospital stay in older patients with cervical and trochanteric hip fracture (HF) (Pearson correlation coefficients)

Parameters	Cervical HF		Trochanteric HF	
	
	r	P	r	P
Age - PTH	0.289	< 0.001	0.209	0.014

Age - eGFR	-0.312	< 0.001	-0.238	0.009

Age - cTnI	0.262	0.002	0.222	0.012

Age - adiponectin	0.245	0.004	0.261	0.004

PTH - calcium	-0.380	< 0.001	-0.180	0.036*

cTnI - eGFR	-0.232	0.008	-0.332	< 0.001

PTH - eGFR	-0.320	< 0.001	-0.386	< 0.001

Haemoglobin - eGFR	0.250	0.002	0.217	0.009

Haemoglobin - albumin	0.348	< 0.001	0.202	0.016

PTH - adiponectin	0.167	0.053*	0.268	0.004

PTH - leptin	0.235	0.005		

PTH - LOS	0.200	0.017		

LOS - phosphate	0.169	0.044*		

Albumin - eGFR	0.244	0.002		

PTH - phosphate			0.199	0.020*

PTH - cTnI			0.397	< 0.001

PTH - adiponectin			0.268	0.004

PTH - resistin			0.193	0.038*

Age - leptin			- 0.301	< 0.001

Age - resistin			0.238	0.009

Leptin - adiponectin			- 0.251	0.006

Leptin - haemoglobin			0.262	0.002

Leptin - CRP			0.226	0.008

Adiponectin - CRP			- 0.205	0.026*

Resistin - eGFR			- 0.301	< 0.001

Resistin - calcium			0.227	0.014

cTnI - haemoglobin			- 0.195	0.028*

eGFR, estimated glomerular filtration rate; cTnI, cardiac troponin I; CRP, C-reactive protein; LOS, length of stay. ^* ^Non-significant correlations when Bonferroni and Sidak adjustments for multiplicity adopted.

In patients with cervical HF, PTH correlated positively with leptin and LOS, and albumin correlated with eGFR. In patients with trochanteric HF, PTH correlated positively with serum phosphate, adiponectin, resistin and cTnI. Only in this group age correlated negatively with leptin and positively with resistin; leptin correlated positively with haemoglobin and negatively with adiponectin and adiponectin correlated negatively with CRP; resistin correlated positively with calcium and negatively with eGFR and cTnI correlated negatively with haemoglobin.

### Hip fracture type and short-term outcomes

Short-term outcomes such as peri-operative myocardial injury (assessed by cardiac troponin I rise), length of hospital stay (LOS), being discharged to RCF as well as in-hospital death did not show significant difference between patients with cervical and trochanteric HF (Table 1). The mean values of the highest post-operative CRP concentrations and the incidence and degree of its elevation also did not differ between groups.

### Hip fracture type and predictors of outcomes

Only a limited number of the 17 clinical and 35 laboratory parameters tested were found associated with risk of poorer outcomes. On univariate analyses these included age, sex, CAD, dementia, ASA ≥ 3, smoking, eGFR, haemoglobin, 25(OH)D, PTH, leptin, adiponectin and resistin levels, but with different impact on specific outcomes by HF type.

Multivariate analyses which included only clinical characteristics (age, sex, residency, above mentioned co-morbidities, ASA score ≥ 3, and smoking) on admission revealed the following significant independent predictors for specific outcomes. *Post-operative myocardial injury (cTnI > 0.06 μg/L)*: in patients with cervical HF age (OR = 1.13; 95% CI 1.05-1.23; p = 0.001) and current smoking (OR = 11.2; 95% CI 1.4-88.3; p = 0.022); in patients with trochanteric HF age (OR = 1.13; 95% CI 1.03-1.24; p = 0.009) and history of CAD (OR = 6.4; 95% CI 1.7-24.2; p = 0.007). *Post-operative marked inflammatory response (CRP > 100 mg/L)*: age (OR = 0.92; 95% CI 0.86-0.98; p = 0.013) in patients with trochanteric HF. *Prolonged LOS (≥ 20 days)*: in cervical HF patients CAD (OR = 4.3; 95% CI 1.5-11.9; p = 0.005) and in trochanteric HF patients age (OR = 1.08; 95% CI 1.02-1.15; p = 0.014). *Need of institutionalization*: in cervical HF age (OR = 1.17; 95%CI 1.05-1.29; p = 0.003), ASA ≥ 3 (OR = 5.0; 95% CI 1.2-21.6; p = 0.031) and dementia (OR = 80.2; 95% CI 5.3-321.1; p = 0.002), in trochanteric HF age (OR = 1.20; 95% CI 1.07-1.35; p = 0.002) and dementia (OR = 6.0; 95%CI 1.2-30.2; p = 0.031). All patients who died in the hospitals had ASA ≥ 3 and were smokers. None of the other clinical variables in multivariate models were independent predictors of in-hospital death.

Further confirmation of important differences between cervical and trochanteric HF patients in respect to factors that influence outcomes has been noted from the analyses of laboratory parameters. In the multivariate models which included 25(OH) D, PTH, haemoglobin, albumin, calcium, phosphate, magnesium, eGFR, leptin, adiponectin and resistin as continuous independent variables, the following factors emerged as significant predictors of poorer outcomes. *Post-operative myocardial injury*: in patients with cervical HF eGFR (OR = 0.97; 95%CI 0.94-0.99; p = 0.007) and in patients with trochanteric HF, PTH (OR = 1.11; 95% CI 1.01-1.23; p = 0.038), 25(OH) D (OR = 0.96; 95% CI 0.92-0.99; p = 0.034), eGFR (OR = 0.95; 95% CI 0.92-0.99; p = 0.007). *Inflammatory response*: 25(OH)D (OR = 0.97; 95%CI 0.95-0.99; p = 0.017) in patients with cervical HF. *Prolonged LOS*: PTH (OR = 1.13; 95%CI 1.01-1.27; p = 0.037) in patients with cervical HF. *Need of institutionalization*: leptin (OR = 0.96; 95% CI 0.92-0.99; p = 0.033) in patients with trochanteric HF. *In-hospital death*: in patients with cervical HF PTH (OR = 1.40; 95% CI 1.04-1.88; p = 0.027) and leptin (OR = 0.81; 95% CI 0.66-0.99; p = 0.040) and PTH (OR = 1.34; 95% CI 1.03-1.73; p = 0.027) in patients with trochanteric HF.

Finally, logistic regression analyses which included both clinical and laboratory parameters as categorical variables (all with p ≤ 0.15 in univariate analyses) were performed to identify independent risk or protection factors for each study short-term outcome (as the dependant variable). For prolonged LOS, need of a permanent RCF and in-hospital mortality postoperative troponin rise and marked CRP elevation were also included in the models. Table 4 shows that independent predictors of specific outcomes differ significantly by HF type. Independent predictors of postoperative myocardial injury in patients with cervical HF were dementia, renal failure and current smoking. In subjects with trochanteric HF this complication was predicted by a history of CAD, secondary hyperparathyroidism and lower leptin levels. Only in trochanateric HF was anaemia on admission predictive for a marked inflammatory response and lower leptinaemia had a "protective effect" (suppressed CRP). Prolonged LOS in cervical HF patients was predicted by presence of CAD and in the trochanteric group by age ≥ 75 years and elevated PTH. In both groups need of institutionalisation was strongly predicted by age ≥ 75 years and pre-existing dementia. Hypovitaminosis D in subjects with cervical HF and secondary hyperparathyroidism in patients with trochanteric HF were also independent predictors of this outcome. The independent predictors of in-hospital death were male sex, elevated PTH and lower leptin levels in patients with cervical HF, and secondary hyperparathyroidism in subjects with trochanteric HF.

**Table 4:** Independent predictors of short-term outcomes in older patients with cervical and trochanteric hip fracture (HF)

Outcome	Predictor	Cervical HF			Trochanteric HF		
		
		OR	95% CI	P	OR	95% CI	P
Postoperative myocardial injury (cTnI > 0.06 μg/L)	CAD				5.9	1.5-22.3	0.010
	
	Dementia	3.3	1.3-7.9	0.010			
	
	Smoker	18.3	2.2-153.8	0.007			
	
	eGFR < 60 ml/min	3.3	1.4-8.0	0.008			
	
	PTH > 6.8 pmol/L				3.7	1.1-12.0	0.031
	
	Leptin < 11.35 ng/ml				4.8	1.1-20.0	0.039

Marked inflammatory response (CRP > 100 mg/L)	Hb < 120 g/L				6.8	1.6-28.8	0.009
	
	Leptin < 11.35 ng/ml				0.5	0.32-0.94	0.034

LOS ≥ 20 days	Age > 75 years				5.8	1.1-30.9	0.040
	
	CAD	4.5	1.5-13.3	0.006			
	
	PTH > 6.8 pmol/L				3.5	1.1-10.9	0.029

Long-term RCF need	Age > 75 years	14.0	1.01-195.1	0.049	10.7	1.6-73.4	0.016
	
	Dementia	40.0	2.8-571.4	0.007	12.9	2.8-59.5	0.001
	
	25(OH)D < 50 nmol/L	10.3	1.8-57.9	0.008			
	
	PTH > 6.8 pmol/L				7.0	1.2-43.1	0.035

In-hospital death	Sex (male)	6.9	1.04-45.6	0.045			
	
	PTH > 6.8 pmol/L	28.4	2.6-306.0	0.006	7.3	2.1-31.9	0.003
	
	Leptin < 11.35 ng/ml	14.3	1.4-99.9	0.025			

Multivariate logistic regression analyses included all variables with P ≤ 0.15 on univariate analyses.

CAD, coronary artery disease; cTnI, cardiac troponin I; CRP, C-reactive protein; eGFR, estimated glomerular filtration rate; RCF, residential care facility.

## Discussion

Our study shows that the socio-demographic, clinical and routine laboratory profiles as well as the incidence of poorer short-term outcomes in the two main HF types are rather similar, but the risk factors for the short-term outcomes are different, likely reflecting the differences in the underlying mechanisms especially PTH and adipocytokines dysregulation.

In our cohort of 761 unselected consecutive HF patients there was no significant difference between the two HF types in respect to gender, residential status, chronic illness, ASA score, ambulating with an assistive device, smoking and alcohol consumption. Our data on demography, comorbidity burden and mortality in older HF patients is comparable to that in previous published studies [43] and consistent with observations that there are no differences between the two HF groups in the type and number of comorbidities and prefracture residence [15]. In our series, statistically significant differences between cervical and trochanteric HF groups were mean age (in the latter group females were older than males) and prevalence of Parkinson's disease (higher in the former group). The first observation is in line with many [7,20,22,44] but not all [15] previous reports; the cause of the second is unclear.

Comparison of a wide range of routine haematologic and biochemical variables including renal, liver and thyroid function tests showed that only mean haemoglobin and albumin levels were associated with HF type, being slightly lower in the trochanteric group.

Much recent attention has focussed on health effects of factors with multiple physiological roles such as vitamin D, PTH and adipocytokines, peptides secreted mainly by white adipose tissue. However the role of anatomical location of HF in these relationships and the potential implications for clinical practice are largely not known.

In accordance with previous reports [45-47], we found a very high prevalence of hypovitaminosis D in both types of HF (77.8% in cervical and 82.1% in trochanteric) and secondary hyperparathyroidism in 30.2% and 41.3% respectively. Trochanteric compared to the cervical HF patients, despite similarly low mean 25(OH) D concentrations, had a 36.5% increase in mean serum PTH level. These results are in agreement with those reported by many other investigators, except Dretakis et al [48] who among 53 older women with HF found that 25(OH)D levels were significantly lower in patients with trochanteric compared to those with cervical HF, while the PTH levels were simular. Another important difference between cervical and trochanteric HF groups in our study is that in the former mean levels of adiponectin and resistin were significantly higher than in the latter.

We found no noteworthy differences between the two HF types in terms of postoperative inflammatory response (assessed by CRP evaluation) and myocardial injury (evaluated by cTnI rise), nor in LOS, percentage of subjects being newly discharged to long-term RCF and in-hospital mortality rate. Data on the associations between HF type and short-term outcomes are sparse and inconsistent, though some previous reports found a longer LOS [29,30] and higher mortality in trochanteric HF [7,15,17,34]. Our results are in line with observations that HF type is not an independent prognostic factor [43,49] and is not associated with LOS [15]. Our findings coupled with the literature data show that prognosis of short-term outcomes based exclusively on HF type is unhelpful.

The major and novel finding in this study is that the risk factor profiles for certain outcomes differ by the HF type. Comparison of outcomes in cervical and trochanteric HFs in principle cannot provide evidence for or against the hypotheses that two fracture types share common pathophysiology. Statistically similar incidence of certain outcomes can arise from different factors, and similar abnormalities may contribute to different outcomes. Our data on Pearson correlation coefficients between selected laboratory parameters, including 25(OH)D, PTH, adipocytokines, age and LOS, documented similarities and differences between the two HF types. In total, there were 10 significant correlations common for both HF types, 4 statistically significant relationships observed only in cervical HF and 13 correlations were seen only in trochanteric HF (after Bonferroni and Sedak adjustments for multiplicity 8, 3 and 8 respectively). Although the full discussion of the reason for these differences is beyond the scope of this paper, the present data suggest that the complex relationships between PTH, adipocytokines, renal function and age in older HF patients are site-specific.

Our multivariate analyses of a variety of laboratory parameters as continuous variables revealed that post-operative myocardial injury in patients with cervical HF was independently predicted only by a decrease in eGFR, while in patients with trochanteric HF also by increased PTH levels and decrease in serum 25(OH) D. Lower leptin levels were independently associated with in-hospital death in the former group and with need of institutionalization in the latter group. In patients with cervical HF, higher PTH levels were predictive of prolonged LOS and in-hospital death, and lower 25(OH) D indicated a marked inflammatory response, whereas in patients with trochanteric HF, PTH was associated with in-hospital mortality but none of the laboratory parameters predicted an inflammatory response or LOS. Increasing age as a continuous variable was a significant independent predictor of post-operative myocardial injury and need of institutionalization in both groups and predicted LOS and inflammatory response (negatively) in patients with trochanteric HF.

To provide practicing physicians with useful prognostic indicators regarding short-term HF outcomes we performed multivariate analyses of clinical and laboratory characteristics as categorical variables. These showed that in models based on HF type, clinical characteristics on admission were predictive of certain unfavourable short-term outcomes indicating that outcomes depend in large part on the preoperative conditions and the risks are HF-site specific. Not surprisingly advanced age (> 75 years), dementia and history of CAD were the most often and easily ascertained clinical predictors of poor outcomes but differ by HF type. Our final models included both clinical and laboratory variables. Although adding laboratory parameters often resulted in a relatively small improvement of overall prediction, the laboratory characteristics yielded useful pathophysiological information concerning certain HF outcomes and potential targets for preventive and therapeutic interventions.

**Figure 1:**
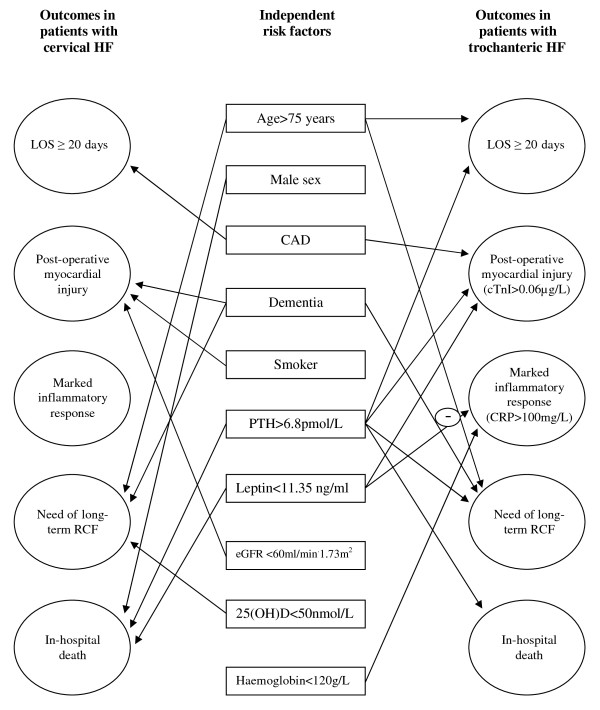
**Independent risk factors for short-term outcomes in older patients with hip fracture by fracture site**. CAD, coronary artery disease; LOS, length of hospital stay; RCF, residential care facility; cTnI, cardiac troponin I; CRP, C-reactive protein.

Among a variety of laboratory parameters examined, independent associations with certain outcomes demonstrated only five analysed as categorical variables (elevated PTH, low leptin, 25(OH)D, eGFR and haemoglobin) with PTH and leptin predicting 4 and 3 outcomes, respectively (Figure 1). Previously we have shown that elevated PTH is a strong independent predictor of poor outcomes in older HF patients [50]. This study importantly adds that the strong independent associations of PTH with certain adverse events are HF-type specific. Secondary hyperparathyroidism predicted in-hospital death in both HF types, but was associated with post-operative myocardial injury, prolonged LOS and need in a long-term RCF only in patients with trochanteric HF. Similarly, in a prior study we found that lower leptin levels indicate poor outcome, specifically myocardial injury and in-hospital death [51]. Our results are in line with a recently published prospective study which found that low leptin was associated with increased cardiovascular events and mortality in patients with stable CAD [52], although others reported contrary observations [53,54]. Patient-related factors (HF type and related mechanisms in our case) are a possible explanation for this discrepancy. The current study found that lower serum leptin is an independent predictor of the myocardial injury in patients with trochanteric HF and in-hospital death in patients with cervical HF.

Our results are in line with the growing evidence that bone mineral density (BMD), mechanical, geometrical and material characteristics of the proximal femur as well as clinical risk factors significantly differ in patients with cervical and trochanteric HF [7,9,15,16,55-57]. Our observations are also supported by the fact that PTH [58-61], leptin and other adipocytokines [62,63]; have differing effects on cortical and trabecular bone, including the hip. Furthermore, recent studies indicate common aetiologies and mechanisms for certain medical illnesses, bone loss and osteoporotic fractures [64,65], and PTH and adipocytokines are important factors acting in bone, cardiovascular, metabolic and kidney diseases [62]. Taken together, these data suggest important differences in metabolic interrelationships and pathogenetic pathways which may simultaneously contribute to HF type, comorbidities and certain outcomes. Whereas additional work will be needed to identify the optimal variables for predicting HF outcomes, our study confirms that the risk factors should be assessed separately for cervical and trochanteric HFs.

Our study based on evaluation of a relatively large number of unselected consecutive and prospectively observed older patients with HF, in whom a wide variety of clinical and laboratory parameters were determined, is the first systematically seeking to clarify the risks of poor short-term outcomes by HF type. Despite this strength, several limitations of the study require consideration. First, because of its cross-sectioned design no inference can be made on causal relationships. Second, no categorization of the causes of death, rise of cTnI and CRP was undertaken and subgroups were not analysed because of the limited number of cases. As each of these outcomes has multifactorial causes and heterogenetic pathophysiology, additional or different risk factors may be found in future studies. Third, all laboratory parameters were measured on admission and thereafter, therefore the possibility that fracture-related stress may affect some metabolic parameters could not be excluded. However, adjustment for urinary cortisol levels normalised for creatinine (not shown) did not modify any of the described associations. We also acknowledge that multiple comparisons may potentiate the significance of multicollinearity phenomena in multivariate regression analysis. However, the variance inflation factor in all prediction models presented in Table 4 was less than 1.7, indicating that the amount of multicollinearity was not significant. Lastly, the results of the study may not be applicable to all elderly HF patients due to the very high prevalence of white Caucasians in our cohort.

## Conclusions

Clinical characteristics and incidence of poorer short-term outcomes in the two main HF types are rather similar but risk factors for certain outcomes are site-specific reflecting differences in underlying mechanisms.

## Competing interests

The authors have no financial or any other kind of personal conflicts with this paper.

## Authors' contributions

AAF initiated the study, developed the study protocol, collected data, coordinated data analysis, reviewed the literature, drafted and reviewed the manuscript. WS performed the statistical analysis and commented on paper drafts. MWD and PNS participated in the conceptualization of the study. All authors have read and approved the final manuscript.
